# Dual Blockade of Lactate/GPR81 and PD-1/PD-L1 Pathways Enhances the Anti-Tumor Effects of Metformin

**DOI:** 10.3390/biom11091373

**Published:** 2021-09-17

**Authors:** Shaomeng Chen, Xiuman Zhou, Xin Yang, Wanqiong Li, Shuzhen Li, Zheng Hu, Chen Ling, Ranran Shi, Juan Liu, Guanyu Chen, Nazi Song, Xianxing Jiang, Xinghua Sui, Yanfeng Gao

**Affiliations:** 1 School of Pharmaceutical Sciences (Shenzhen), Sun Yat-sen University, Shenzhen 518107, China; chenshm56@mail2.sysu.edu.cn (S.C.); zhouxm36@mail.sysu.edu.cn (X.Z.); yangx295@mail2.sysu.edu.cn (X.Y.); liwq67@mail2.sysu.edu.cn (W.L.); lishzh35@mail2.sysu.edu.cn (S.L.); huzh37@mail2.sysu.edu.cn (Z.H.); lingch6@mail2.sysu.edu.cn (C.L.); liuj756@mail.sysu.edu.cn (J.L.); chengy239@mail.sysu.edu.cn (G.C.); 2 School of Life Sciences, Zhengzhou University, Zhengzhou 450001, China; srr@gs.zzu.edu.cn; 3 School of Pharmaceutical Sciences, Sun Yat-sen University, Guangzhou 511400, China; songnz@mail2.sysu.edu.cn (N.S.); jiangxx5@mail.sysu.edu.cn (X.J.)

**Keywords:** metformin, GPR81, lactate, cancer immunotherapy, PD-1/PD-L1 blockade

## Abstract

Metformin is a widely used antidiabetic drug for cancer prevention and treatment. However, the overproduction of lactic acid and its inefficiency in cancer therapy limit its application. Here, we demonstrate the synergistic effects of the lactate/GPR81 blockade (3-hydroxy-butyrate, 3-OBA) and metformin on inhibiting cancer cells growth in vitro. Simultaneously, this combination could inhibit glycolysis and OXPHOS metabolism, as well as inhibiting tumor growth and reducing serum lactate levels in tumor-bearing mice. Interestingly, we observed that this combination could enhance the functions of Jurkat cells in vitro and CD8^+^ T cells in vivo. In addition, considering that 3-OBA could recover the inhibitory effects of metformin on PD-1 expression, we further determined the dual blockade effects of PD-1/PD-L1 and lactate/GPR81 on the antitumor activity of metformin. Our results suggested that this dual blockade strategy could remarkably enhance the anti-tumor effects of metformin, or even lead to tumor regression. In conclusion, our study has proposed a novel and robust strategy for a future application of metformin in cancer treatment.

## 1. Introduction

Despite considerable efforts and investments in the development of new cancer drugs, only 5% of them were approved for clinical therapeutics [1]. The substantial costs and slow pace of new drug discovery and development have provoked the exploration of new uses for approved drugs that are outside the scope of their original medical indication [2]. Compared with developing new drugs from scratch, the repurposing of existing drugs might potentially lower overall development costs and shorten development timelines.

Among the repurposed drugs, metformin is a promising candidate for cancer therapy. As a first-line drug for type 2 diabetes, metformin showed promising preventive effects on cancer [3,4,5]. Diabetics receiving metformin had a lower incidence of cancer than other patients [6,7]. Moreover, it has been found that metformin can directly suppress tumor growth by the activation of AMP activated protein kinase (AMPK) pathway, cell cycle arrest, and metabolic disorder [8,9,10]. Additionally, through promoting the secretion of tumor necrosis factor-α (TNF-α) and interferon γ (IFN-γ), metformin could significantly boost the anti-tumor activity of CD8^+^ T cells to indirectly retard tumor progression [11]. However, only high doses of metformin could impede tumor growth [12]. Unfortunately, high doses of metformin can lead to lactic acidosis and elevate intra-tumoral lactate levels [13,14]. Therefore, the application of metformin is a double-edged sword in cancer therapy, and it is crucial to employ the anti-tumor effects of metformin while reducing the undesired side effects caused by lactate.

Lactate, as the metabolite from cancer cells, facilitates cancer progression and metastasis [15,16,17]. Considerable studies have shown that excessive lactic acid induces a decrease in pH of the tumor microenvironment, which restrains the mobility and the cytotoxic function of T cells and even leads to apoptosis [18,19]. Lactate transmits pro-tumor signals through its receptor G-Protein Coupled Receptor 81 (GPR81), which is overexpressed in various types of cancer cells. The knockout of GPR81 can lower the sensitivity of tumor cells toward lactate, which allows to suppress tumor proliferation and metastasis [15]. Additionally, the activation of GPR81 mediated by lactate can also increases the expression of PD-L1 in tumor cells, to reduce IFN-γ secretion and the cell viability of co-cultured Jurkat cells [20]. A putative GPR81 antagonist, 3-hydroxy-butyrate (3-OBA) can be used to block the lactate/GPR81 pathway and protect cells from glucose deprivation [21]. Therefore, its application together with metformin will bring the opportunity to enhance the anti-tumor effects and reduce the side effects of metformin on lactate production.

Here, to improve the anti-tumor effect of metformin and reduce lactic acidosis, we sought to investigate whether blocking the lactate/GPR81 pathway could enhance the anti-tumor effects of low concentrations of metformin. Furthermore, we investigated the influence of the PD-1/PD-L1 blockade on the overall therapeutic performance of the combinational therapy. The results of this study demonstrate new evidence of this therapeutic mechanism and provide an effective strategy for anti-tumor application of metformin.

## 2. Materials and Methods

### 2.1. Cell Culture and Reagents

Human colon cancer cell lines HT29 and RKO, murine colon cancer cell line CT26, and the human T-lymphoblastic Jurkat cell line were purchased from ATCC and conserved by our laboratory. All cells were cultured in Dulbecco’s Modified Eagle Medium (DMEM) (Gibco, Grand Island, NY, USA) with 10% fetal bovine serum (FBS) (Sigma-Aldrich, St. Louis, MO, USA), supplemented with 100 μg/mL streptomycin (Solarbio, Beijing, China) and 100 U/mL penicillin (Solarbio, Beijing, China). Cells were cultured at 37 °C in an incubator with 5% CO_2_. Metformin (purity > 97.0%, Sangon Biothech, Shanghai, China), and 3-OBA (Sigma-Aldrich, St. Louis, MO, USA) was purchased.

### 2.2. TIMER Analysis, RNA Isolation and Quantitative Real-Time Polymerase Chain Reaction (PCR)

The Diff Exp module in the TIMER website (https://cistrome.shinyapps.io/timer/, accessed on 27 September 2019) was used to analyze the expression of GPR81 between tumor and normal tissue. Total RNA was extracted using RNA Isolation Kits (Vazyme, Nanjing, China) and then reversely transcribed into cDNA using HiScript III 1st Strand cDNA Synthesis Kits (Vazyme, Nanjing, China) according to the manufacturer’s instructions. We obtained the primer sequences for the targets from PrimerBank (https://pga.mgh.harvard.edu/primerbank/, accessed on 20 November 2019 and 5 January 2021), and verified their specificity in Primer-BLAST (https://www.ncbi.nlm.nih.gov/tools/primer-blast/, accessed on 20 November 2019 and 5 January 2021). The primer sequences used in experiments were synthesized by Sangon Biothech (Shanghai, China) and are listed in Table S1. After mixing the cDNA with SYSB qPCR Master-Mix (Vazyme, Nanjing, China) and setting the condition of the PCR reaction as follows: predegeneration at 95 °C for 3 min, 40 cycles of denaturation at 95 °C for 10 s, and extension at 60 °C for 30 s; melt curves were obtained at 95 °C for 15 s, 60 °C for 60 s, and 95 °C for 15 s. cDNA expression was monitored in a LightCycler 96 system (Roche, Basel, Switzerland).

### 2.3. Cell Growth Assay

HT29, RKO and CT26 cells were seeded in 96-well plates with 8 × 10^3^, 4 × 10^3^ and 3 × 10^3^ cells per well, respectively, for 24 h. Then cells were treated with different concentration of metformin (5 mM, 15 mM, and 45 mM) and 3-OBA (0.1 mM, 1 mM, 10 mM, 15 mM, and 20 mM) for 48 h. Next, 20 μL of Cell Counting Kit-8 (CCK-8) (Beyotime, Shanghai, China) was added into each well and incubated for 2 h at 37 °C. Absorbance at 450 nm was determined by the microplate reader (PerkinElmer, Waltham, MA, USA). To evaluate the combinational effect of metformin and 3-OBA, we used the King’s method to calculate the Q value [22]. The Q value of 5 mM metformin and 15 mM 3-OBA was more than 1.15 and was the largest. Thus, we finally selected the combination of 5 mM metformin and 15 mM 3-OBA, with their obvious synergistic effect, to perform the studies.

### 2.4. Cell Apoptosis Assay

HT29 cells were seeded in 24-well plates with 5 × 10^4^ cells per well for 24 h. Then cells were treated with 15 mM 3-OBA, 5 mM metformin, and the combination. After 48 h, the cells were harvested and resuspended with Phosphate Buffered Saline (PBS) buffer. According to the manufacturers’ instructions of apoptosis assay kit (Beyotime, Shanghai, China), we added 195 μL Annexin V-FITC binding buffer, 5 μL Annexin V-fluorescein isothiocyanate (FITC), and 10 μL propidium iodide (PI) to 1 × 10^5^ cells. Following a gentle vortex, the cells were incubated at room temperature for 20 min in the dark. Finally, flow cytometry was used to monitor fluorescence intensity.

### 2.5. Western Blot Analysis

After being treated with 15 mM 3-OBA, 5 mM metformin and the combination, HT29 cell were collected and lysed. The lysates were subjected to 8% polyacrylamide gels with SDS. Then the protein was transferred onto polyvinylidene fluoride membranes. The membranes were incubated by primary antibodies against β-actin (3700S, CST, 1:1000), GPR81 (A20321, ABclonal, 1:1000), mTOR (2983S, CST, 1:1000), and p-mTOR (5536S, CST, 1:1000), and recognized with secondary antibodies.

### 2.6. Glucose Uptake Assay

After HT29 and RKO cells were treated with 5 mM metformin and 15 mM 3-OBA for 48 h, the cells were incubated in glucose-free medium with 2 mM L-glutamine for 3 h. Then, 100 μM 2-Deoxy-2-[(7-nitro-2,1,3-benzoxadiazol-4-yl) amino]-D-glucose (2-NBDG) was added to the incubate for 30 min. Subsequently, the cells were collected and the fluorescence intensity was analyzed by flow cytometry.

### 2.7. Glycolysis and Mitochondrial Stress Test

HT29 and RKO cells were seeded into an XF24 culture microplate (Seahorse Bioscience, Billerica, MA, USA) at densities of 4 × 10^4^ and 1 × 10^4^ cells per well, respectively. After incubating overnight, cells were treated with 5 mM metformin, 15 mM 3-OBA, or the combination for 24 h. Then cells were rinsed twice with assay medium and placed in a non-CO_2_ incubator for 45 min. Meanwhile, glucose, oligomycin (OM), 2-deoxy glucose (2-DG), carbonyl cyanide-4 (trifluoromethoxy) phenylhydrazone (FCCP), and rotenone/antimycin A were diluted with the assay medium to specified concentrations. Finally, these reagents were loaded into the sensor cartridge and the plate was placed into Seahorse XFe24 Extracellular Flux Analyzers (Seahorse Bioscience, Billerica, USA) to monitor the extracellular acidification rate (ECAR) and the oxygen consumption rate (OCR).

### 2.8. Jurkat Cell Proliferation and Function Analysis

Under the stimulation of 1 μg/mL anti-human CD3 antibody (OKT3, eBioscience, San Diego, CA, USA) and 0.5 μg/mL anti-human CD28 antibody (CD28.2, eBioscience, San Diego, CA, USA), Jurkat cells were treated with 5 mM metformin, 15 mM 3-OBA, or the combination for 48 h. Alternatively, HT29 cells were treated with 5 mM metformin, 15 mM 3-OBA, or the combination for 48 h. Then culture supernatant was taken out separately to treat Jurkat cells for 48 h. A CCK8 kit was used to determine cell proliferation, or after pretreating cells with protein transport inhibitor for 4 h, cells were fixed and permeabilized for staining with P-phycoerythrin (PE) anti-human IFN-γ antibody (4S.B3, eBioscience, San Diego, CA, USA) and allophycocyanin (APC) anti-human IL-2 antibody (MQ1-17H12, eBioscience, San Diego, CA, USA). To distinguish the negative and positive cells, we used the matched isotype control or fluorescence minus one (FMO) control to define their boundaries.

### 2.9. Xenograft Tumor Growth

Animal experiments were approved by the Ethics Committee of Sun Yat-Sen University (SYSU-20200437) and performed complying to the national and institutional guidelines. Female BALB/c nude mice and female BALB/c mice (18–22 g) were purchased from Guangdong Medical Laboratory Animal Center. 1 × 10^7^ HT29 and 2 × 10^5^ CT26 cells were subcutaneously injected into the right flanks of BALB/c nude mice or the BALB/c mice, respectively. Tumor volumes were measured using a digital caliper every other day, and calculated as V (mm^3^) = 0.5 × length × width × height. When tumor volumes reached 40–80 mm^3^, mice were randomly divided into groups. Metformin (200 mg/kg), 3-OBA (75 mg/kg) and the combination of 3-OBA and metformin with the same dosage were intraperitoneally injected into mice every day. PD-1/PD-L1 blocking peptide C8 (2 mg/kg) [23] and the negative control normal saline were injected paratumorally every other day. Body weights were recorded every other day.

### 2.10. Serum Lactate Measurement

According to the manual of Lactate Assay Kit (Solarbio, Beijing, China), 100 µL of serum was sampled from mice and added into 1 mL of extracting solution I. After being centrifuged at 12,000× *g* at 4 °C for 10 min, 0.8 mL of the supernatant was then withdrawn. After 150 µL of extracting solution II was added, the samples were centrifuged at 12,000× *g* for 10 min. Then, the supernatant was harvested and measured.

### 2.11. Flow Cytometry

After treatment with metformin or 3-OBA, Jurkat cells were collected and stained with PE anti-human PD-1 antibody (MIH4, eBioscience, San Diego, CA, USA). The expression of PD-1 was evaluated using flow cytometry. Tumor tissues were removed from the mice, minced, and digested by collagenase IV (Invitrogen, Carlsbad, CA, USA) and Dnase I (Sigma-Aldrich, St. Louis, MO, USA) at 37 °C in a shaker for 40 min. Spleen and draining lymph nodes were ground and filtrated with a 70 μm filter. All samples were stained with FITC anti-mouse CD45 antibody (30-F11, eBioscience, San Diego, CA, USA), PerCP-eFluor 710 anti-mouse CD3 antibody (17A2, eBioscience, San Diego, CA, USA), Brilliant Violet 421 anti-mouse CD8 antibody (53–6.7, BioLegend, San Diego, CA, USA), and APC anti-mouse PD-1 antibody (RMP1-30, eBioscience, San Diego, CA, USA) for 30 min. After fixation and permeabilization, the cells were stained by PE anti-mouse IFN-γ antibody (XMG1.2, eBioscience, San Diego, CA, USA) for 30 min. The fluorescence intensity was analyzed by flow cytometry, and the matched isotype control was applied to distinguish the negative and positive cells.

### 2.12. Statistical Analysis

Results are presented as means ± standard deviation and were analyzed by a Student’s *t*-test or one-way analysis of variance (ANOVA). *p* < 0.05 was considered statistically significant.

## 3. Results

### 3.1. The Anti-Tumor Efficacy of Metformin and 3-OBA on Colorectal Cancer Cells

**Figure 1:**
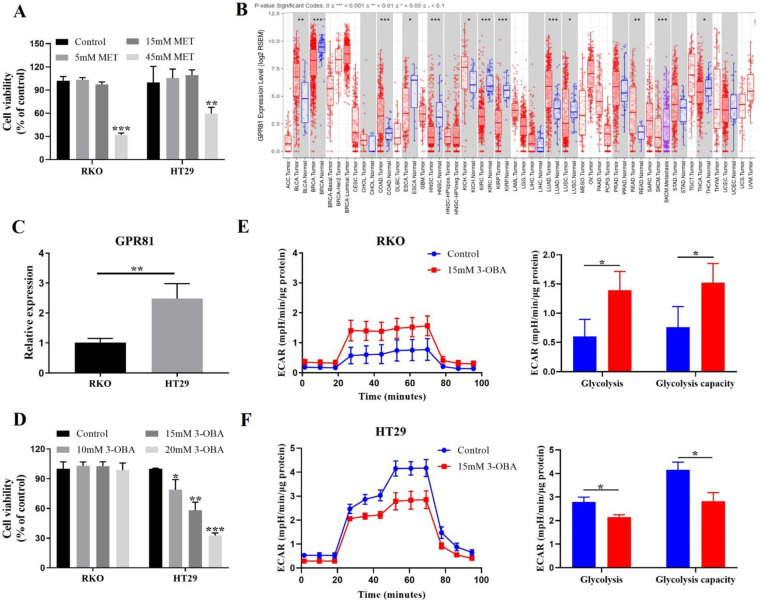
Effects of metformin and 3-OBA on cell proliferation and metabolism. (**A**) RKO and HT29 cells were treated with various concentrations of metformin (5 mM, 15 mM, 45 mM) for 48 h. Cell viability was measured by CCK-8 assay. (**B**) GPR81 expression of a wide variety of cancers in TIMER. (**C**) Relative mRNA expression of GPR81 in RKO and HT29 cells. (**D**) RKO and HT29 cells were treated with different concentrations of 3-OBA (10 mM, 15 mM, 20 mM) for 48 h. Cell viability was measured by CCK-8 assay. (**E**,**F**) Extracellular acidification rate (ECAR) of RKO (**E**) or HT29 (**F**) cells pretreated with 15 mM 3-OBA was detected in the presence of glucose, oligomycin (OM), and 2-deoxy glucose (2-DG). Representative histograms of at least three independent experiments are shown. * *p* < 0.05, ** *p* < 0.01, *** *p* < 0.001.

The cell growth assay showed that 45 mM metformin could significantly inhibit the proliferation of human colon cancer cell lines, HT29, and RKO cells, but 5 mM and 15 mM metformin could not (Figure 1A). As reported elsewhere, the effective in vitro concentration of metformin was very high, usually between 10 and 40 mM [13,14]. Thus, we selected 5 mM metformin to perform the studies. Considering that metformin may result in lactic acidosis to promote cancer progression, we performed a glycolysis-stress test to monitor glycolysis. As shown in Supplementary Figure S1A,B, metformin elevated glycolysis, suggesting that metformin can indeed promote the lactic acid production of cancer cells.

Since excessive lactate could weaken the anti-proliferation activity of metformin, it is important to interrupt lactate/GPR81 signaling. Firstly, we exploited TIMER [24], a web server, to analyze the expression of GPR81 in multiple cancers. It was found that GPR81 was highly expressed in colorectal cancer compared with normal tissue (Figure 1B). The results of quantitative real-time PCR (Figure 1C) and western blot (Supplementary Figure S1C) showed that GPR81 expression was significantly higher in HT29 cells than in RKO cells. 3-OBA could inhibit the proliferation of HT29 cells, but not RKO cells (Figure 1D; Supplementary Figure S1D,E). Recently, it has been revealed that silencing GPR81 could suppress the expression of lactate transporters and monocarboxylate transporters (MCTs) [15]. Similarly, 3-OBA could inhibit MCT1 and MCT4 expression in HT29 cells with high GPR81 expression levels, but not in RKO cells with low expression of GPR81 (Supplementary Figure S1F,G). These data illustrate that GPR81 may be associated with glycolysis. We monitored glycolysis, and discovered that 3-OBA inhibited the glycolysis of HT29 cells (Figure 1F), while improving that of RKO cells (Figure 1E). Therefore, 3-OBA has an inhibitory effect in the proliferation and glycolysis of colorectal cancer cells with high GPR81 expression, suggesting that 3-OBA can reduce the lactate levels and enhance the anti-tumor effects of metformin.

### 3.2. The Synergistic Effects of Metformin and 3-OBA on Cell Growth and Lactate Metabolism

**Figure 2:**
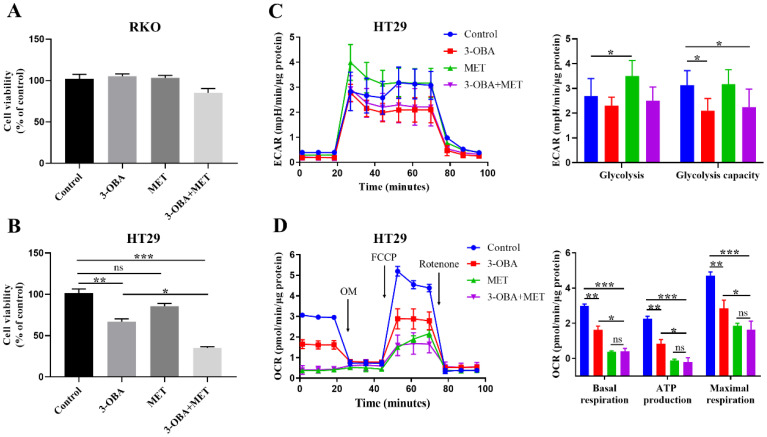
The combination of 3-OBA and metformin inhibits the cell proliferation and lactate metabolism of HT29. (**A**,**B**) RKO (**A**) or HT29 (**B**) cells were treated with 15 mM 3-OBA, 5 mM metformin, and the combination, then cell viability was determined by CCK-8 assay. (**C**,**D**) Extracellular acidification rate (ECAR) (**C**) or oxygen consumption rate (OCR) (**D**) of HT29 cells pretreated with 15 mM 3-OBA, 5 mM metformin, and the combination. The OCR were detected in the presence of oligomycin (OM), carbonyl cyanide-4 (trifluoromethoxy), phenylhydrazone (FCCP), and rotenone. Representative histograms of at least three independent experiments were shown. * *p* < 0.05, ** *p* < 0.01, *** *p* < 0.001; ns represents no significant difference.

Next, we determined whether the anti-tumor effect of metformin was elevated by the combination through a lactate/GPR81 pathway blockade. The results showed that the combination of 3-OBA and metformin had no effects on the proliferation of RKO cells (Figure 2A), but inhibited the proliferation of HT29 cells more significantly than metformin or 3-OBA alone (Figure 2B). As shown in Supplementary Figure S2A, the combination had no effect on cell apoptosis. Similar anti-proliferation effect was shown in the murine colon cancer cell line CT26 (Supplementary Figure S2B). The results of ECAR showed that glycolysis in HT29 cells was blocked by 3-OBA and the combination (Figure 2C). The reduction of lactate production would in turn promote oxidative phosphorylation (OXPHOS), we thus used a mito-stress test to measure OXPHOS. Surprisingly, 3-OBA and the combination also inhibited OXPHOS in addition to inhibiting glycolysis (Figure 2D). Therefore, when combined with 3-OBA, the anti-proliferation effect of metformin was enhanced and lactate metabolism was suppressed.

To investigate the mechanism of glycolysis suppression, we used a 2-NBDG fluorescent probe to detect whether glucose uptake, as the first step of glucose metabolism, was affected. It was observed that glucose uptake was not impacted by 3-OBA, but was significantly increased in the combinational treatment (Supplementary Figure S2C). This observation indicates that the glycolysis inhibiting effects of the combined treatment was not due to glucose uptake. Then, we investigates the expression of metabolic enzymes related to glycolysis. The results showed that metformin down-regulated the expression of hexokinase 2 (HK2), but it was up-regulated by the combination. All three treatments could elevate the expression of aldose 1-epimerase (GALM). Among these enzymes, the expressions of phosphoglycerate mutase 1 (PGAM1), phosphoglycerate mutase 2 (PGAM2), and alpha-enolase (ENO1), located on the upstream of lactate production and OXPHOS, were significantly decreased by treatment with the combination, which might result in glycolysis inhibition and reduced lactate production (Supplementary Figure S2D,E), and might lead to the increase of glucose uptake. The mammalian targets of rapamycin (mTOR) and GPR81 play a vital role in cell metabolism [25,26]. Thus, we detected their protein levels and found that the combined treatment could significantly reduce the expression of GPR81 and mTOR phosphorylation (Supplementary Figure S2F), which may collectively result in the significant inhibition of cell proliferation and metabolism.

### 3.3. 3-OBA Could Enhance the Anti-Tumor Effects of Metformin

**Figure 3:**
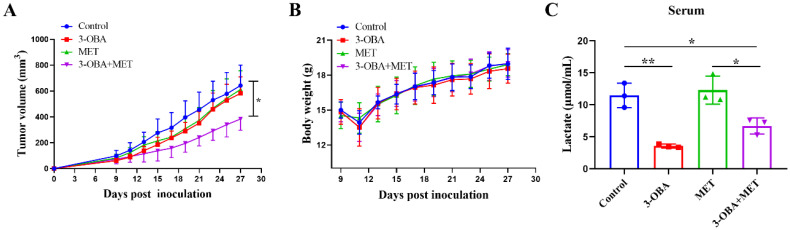
The combination of 3-OBA and metformin inhibits HT29 tumor cell growth in vivo. BALB/c nude mice (*n* = 6) were intraperitoneal injected with 75 mg/kg of 3-OBA, 200 mg/kg of metformin, and the combination, (**A**) tumor volumes and (**B**) body weights were measured every other day. (**C**) Lactate concentration in serum after different treatments. * *p* < 0.05, ** *p* < 0.01.

In the human colon cancer HT29 cell bearing tumor model, the tumor volume was significantly reduced with the combinational treatment of 3-OBA and metformin (Figure 3A). There was no significant difference in body weight between the groups (Figure 3B). High levels of lactate in serum were associated with poor prognosis and reduced overall survival of various cancers [27,28]. We evaluated the serum lactate levels in tumor-bearing mice. The results showed that 3-OBA and the combinational treatment reduced lactic acid levels in serum (Figure 3C).

### 3.4. T Cell Function Could Be Elevated by Metformin and 3-OBA

**Figure 4:**
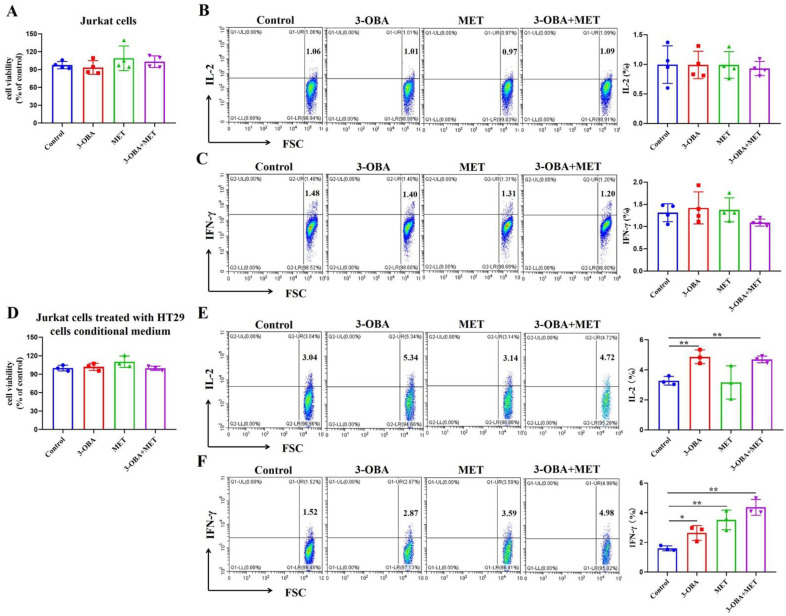
Effects of the combination of 3-OBA and metformin in the proliferation and functions of Jurkat cells. After treatment with 15 mM 3-OBA, 5 mM metformin, or the combination, (**A**) cell viability and (**B**,**C**) the secretions of IL-2 (**B**) or IFN-γ (**C**) by Jurkat cells were measured. After treatment with the conditional medium from HT29 cells treated by 3-OBA, metformin, or their combination, (**D**) cell viability and (**E**,**F**) the secretions of IL-2 (**E**) or IFN-γ (**F**) by Jurkat cells were monitored. Representative histograms of at least three independent experiments were shown. * *p*< 0.05, ** *p* < 0.01.

Since the combinational treatment of 3-OBA and metformin affected the proliferation of tumor cells, we next explored whether the combination might also inhibit T cells, a class of tumor-killing cells. Jurkat cells were treated with the combination, and we found that proliferation (Figure 4A) and the ability to secrete IL-2 and IFN-γ (Figure 4B,C) were unchanged. To investigate whether low glycolysis-mediated lactate level reduction after the combination treatment could promote T cell growth or enhance T cell cytotoxicity, the conditional medium from HT29 cells treated by 3-OBA, metformin, or their combination, was subsequently used to treat Jurkat cells. Although there was no significant difference in the proliferation of Jurkat cells between each group (Figure 4D), the secretion of IL-2 was promoted by the 3-OBA group and the combination group (Figure 4E). Furthermore, all three treatment groups improved the levels of IFN-γ in Jurkat cells, and the secretion of IFN-γ in the combination group was slightly higher than that in the metformin group (Figure 4F). These results suggest that the combination could promote the anti-tumor activity of T cells.

The expression of the immune checkpoints (such as PD-1) in T cells is associated with their cytotoxic function [29,30]. Flow cytometry results showed that metformin down-regulated PD-1 expression of Jurkat cells, while 3-OBA up-regulated its expression. And there was no significant difference in PD-1 expression between the combination and the control (Supplementary Figure S3A). Thus, metformin alone may reduce patients’ response to PD-1-blockade-based immunotherapy, which could be overcome in combination with 3-OBA.

### 3.5. The Combination of Metformin and 3-OBA Could Activate T Cell in Vivo

**Figure 5:**
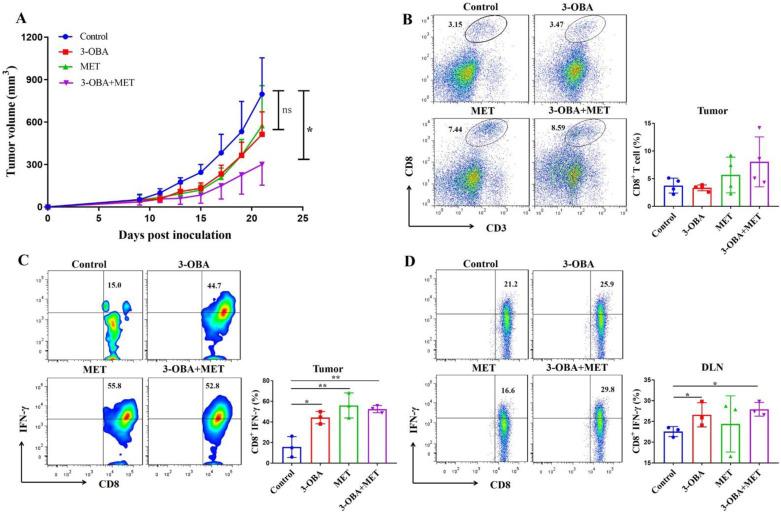
The combination of 3-OBA and metformin could suppress CT26 tumor cell growth and active CD8^+^ T cells. BALB/c mice (*n* = 6) were treated with 75 mg/kg of 3-OBA, 200 mg/kg of metformin, and the combination every day. (**A**) Tumor volumes. (**B**) The ratio of intra-tumoral CD8^+^ T cells. (**C**) The proportion of IFN-γ^+^ CD8^+^ T cells in tumors (**D**) and in the draining lymph nodes. * *p* < 0.05, ** *p* < 0.01. ns denotes no significant difference.

As shown in Figure 5A, the combination treatment significantly inhibited tumor growth in CT26-bearing mice, but metformin or 3-OBA alone did not. Although there was no statistical difference compared with the control group, the percentage of CD8^+^ T cells after combinational treatment did increase (Figure 5B). We found that the IFN-γ levels of intra-tumoral CD8^+^ T cells in all three treatment groups were significantly increased compared with the control group (Figure 5C). Metformin increased the IFN-γ production of CD8^+^ T cells in the spleen, but no significant difference was found in other groups (Supplementary Figure S3B). In the draining lymph nodes, 3-OBA alone and the combination significantly elevated the IFN-γ proportion of CD8^+^ T cells, but metformin did not (Figure 5D). Considering that the function of CD8^+^ T cells in the draining lymph node usually reflects the antitumor immune response inside the tumor, although there was no significant difference in the infiltration of the CD8^+^ T cells, our results indicate that metformin combined with 3-OBA could elevate CD8^+^ T cell functions and thus enhance anti-tumor efficacy.

### 3.6. PD-1 Blockade Could Further Improve the Antitumor Efficacy of Metformin/3-OBA Combination Treatment

**Figure 6:**
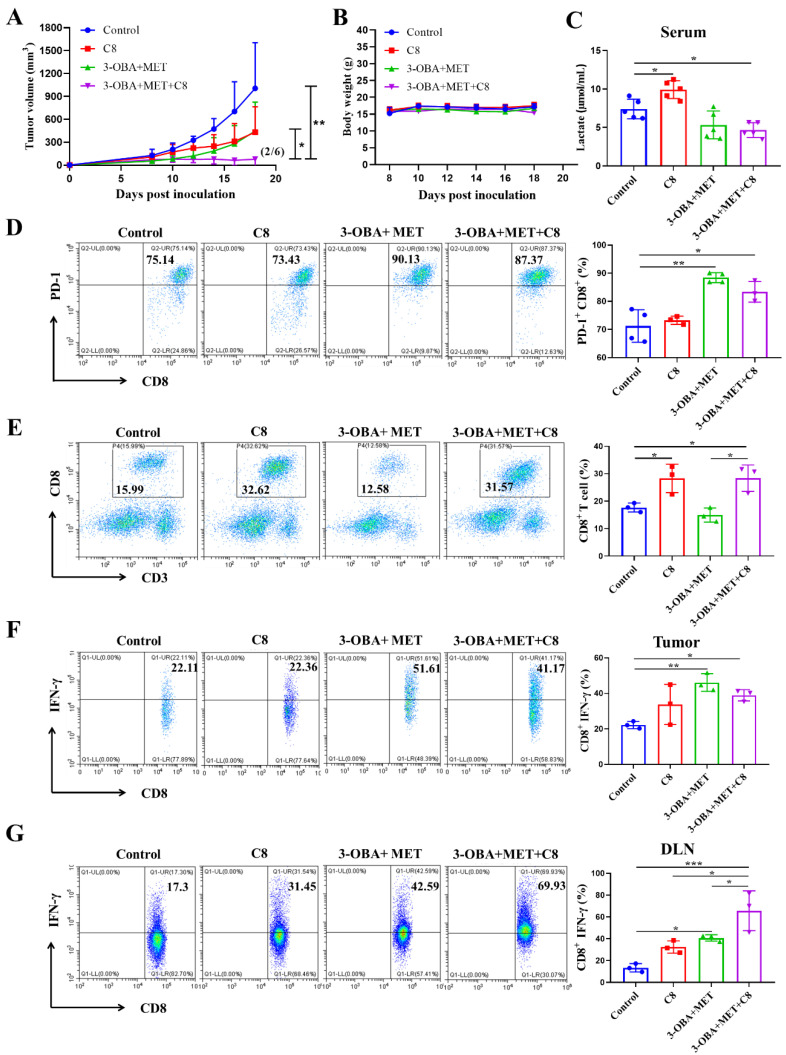
C8 could enhance the anti-tumor effects of the combination of 3-OBA and metformin in vivo. (**A**) Tumor volumes after different treatments. In the combination treatment of 75 mg/kg 3-OBA, 200 mg/kg metformin, and 2 mg/kg C8, tumors of two mice regressed (*n* = 6). (**B**) Body weights. (**C**) Serum lactate in mice. The effects of the combination of 3-OBA, metformin, and C8 on (**D**) PD-1 expression of CD8^+^ T cells, (**E**) intra-tumoral CD8^+^ T cell proportions, (**F**) the ratio of IFN-γ^+^ CD8^+^ T cells in tumors, (**G**) and in the draining lymph nodes. (*n* = 3) * *p* < 0.05, ** *p* < 0.01, *** *p* < 0.001.

To further enhance the antitumor effects, combined with PD-1 blockade may be an appropriate choice. The combination of 3-OBA and metformin was found to reduce the production of lactic acid, and recovered the inhibitory effect of metformin on PD-1 expression. This evidence indicated that the antitumor effects of metformin would be further enhanced by a PD-1 blockade. Here, cyclic peptide C8 was used as a PD-1/PD-L1 inhibitor, as developed by our group previously [23]. In CT26 mouse models, the antitumor effect of C8 combined with metformin and 3-OBA was significantly greater than that of the metformin and 3-OBA combination group and the C8 alone group (Figure 6A). All treatments had no influence on the body weight of the mice, ensuring their safety profiles (Figure 6B). As shown in Figure 6C, serum lactate was increased after C8 treatment, but lower than the control after treatment with the combination of C8, metformin, and 3-OBA. Interestingly, it was observed that the combination of 3-OBA and metformin dramatically up-regulated the expression of PD-1 in intra-tumoral CD8^+^ T cells in vivo, which provided a theoretical basis for the combination PD-1 blockade. Although C8 alone could not affect PD-1 expression, it could be upregulated when combined with 3-OBA and metformin (Figure 6D).

We next determined whether combining the PD-1 blockade was more effective at improving the cytotoxic effect of T cells. It was found that after combination with C8, the proportion of CD8^+^ T cells in tumor tissue was observably elevated compared with the combination of 3-OBA and metformin (Figure 6E). Meanwhile, the addition of C8 significantly elevated the ratio of CD8^+^ IFN-γ^+^ T cells in tumor compared with the control (Figure 6F). These results revealed that the combination of metformin, 3-OBA, and C8 could fully exert the proliferation capacity and cytotoxic activity of CD8^+^ T cells against cancer. Although C8 combined with metformin and 3-OBA did not increase the proportion of CD8^+^ IFN-γ^+^ T cells in the spleen (Supplementary Figure S3C), its ratio in the lymph nodes was significantly increased compared to the C8 group and the combination group of metformin and 3-OBA (Figure 6G). Therefore, the dual blockade of lactate/GPR81 and the PD-1/PD-L1 pathway augments the function of CD8^+^ T cells and prominently enhances the anti-tumor efficacy of metformin.

## 4. Discussion

Drug repurposing is a promising strategy to discover ‘new’ anti-tumor agents from existing ones with full medical specification [31]. Metformin, a commonly prescribed drug for type 2 diabetes, has a proven therapeutic effect in cancer prevention and the reduction of cancer morbidity and mortality [32,33,34]. However, the effective dose of metformin on cancer therapy is often too high, thus inducing the accumulation of lactate and presenting a risk of lactic acidosis [12]. Here, we confirmed that low concentrations of metformin had no anti-proliferative effect on tumor cells, but elevated glycolysis levels to produce excessive lactate. The results of metabolomic analysis showed that metformin could elevate lactate levels [35]. A recent study demonstrated that excessive lactate could lower anti-tumor efficacy of chemotherapeutic agents such as doxorubicin and paclitaxel [36]. Therefore, the production of plethoric lactate may be a key factor to trigger the low anti-tumor efficacy of metformin. GPR81 is a receptor of lactate and mediates the signal transduction of extracellular lactate [15]. Thus, whether blocking lactate/GPR81 pathway can increase the anti-tumor efficacy of metformin should be determined. 

Our results showed that 3-OBA, a GPR81 antagonist, remarkably enhanced the anti-tumor effects of metformin. Moreover, the combination of 3-OBA and metformin was able to lower glycolysis levels and reduce serum lactate levels. Serum lactate is an important prognostic biomarker for cancer patients [37,38], thus its reduction represents a remarkable anti-tumor effect of the combination. For maintaining the physiological functions of tumor cells, metabolic plasticity would shift from glycolysis to OXPHOS [39]. Moreover, metabolic rewiring between glycolysis and OXPHOS is a crucial factor to drive drug resistance [40]. Intriguingly, the combination did not increase the levels of OXPHOS, but decreased them. These results showed that the combination might reduce the chance of chemoresistance.

Finding the tumor inhibitory effects governed by the combination of lactate/GPR81 pathway blockade and metformin, we hypothesized whether it might also impair T cells. We found that the combination could significantly enhance IFN-γ secretion of CD8^+^ T cells in tumors and the draining lymph nodes. Similarly, a recent study has also demonstrated that the IFN-γ secretion of Jurkat cells increased after co-culturing with GPR81-expression cancer cells, as compared to the cells without GPR81 expression [41]. However, the number of intra-tumoral CD8^+^ T cells remained the same. To further enhance the anti-tumor activity of T cells, we combined PD-1/PD-L1 blockade therapy together with the lactate/GPR81 pathway blockade and metformin. Thrillingly, we observed that the additional adoption of PD-1/PD-L1 blockade therapy inhibited tumor growth even more significantly and induced tumor regression, albeit while noting that it also increased the quantity of intra-tumoral CD8^+^ T cells. Furthermore, it was found that the proportion of IFN-γ^+^ CD8^+^ T cells in the draining lymph nodes was elevated remarkably. 

Although the combinational application of metformin and a PD-1/PD-L1 blockade has been reported for cancer therapy, it did not promote the objective response rate (ORR), nor offer a survival benefit [42,43,44]. Metformin indeed suppresses PD-1 and PD-L1 expression [45,46,47], which lowers patients’ response to PD-1/PD-L1 blockade therapy [48]. Therefore, metformin in combination with the PD-1/PD-L1 blockade therapy is inadequate to cause a permanent impairment of tumor cells. In this study, it was interesting to find that when combined with 3-OBA, PD-1 expression was restored to a level even higher than that of the control. In addition, a recent study has illustrated that after treatment with an immune checkpoint inhibitor, the mice bearing glycolysis-low tumors survived longer and had lower metastasis rates than mice with highly glycolytic tumors [49]. These data showed that the increase of PD-1 expression and the decrease of glycolysis induced by blocking the lactate/GPR81 pathway could enhance the combinational anti-tumor effects of metformin and the PD-1/PD-L1 blockade. 

In the present study, a combinational therapy of 3-OBA and metformin demonstrated a significant anti-tumor effect on the HT29 cell line and a PD-1-responsive CT26 tumor model. However, whether the combination can improve the response to the treatment of PD-1/PD-L1 blockade in clinical settings remains uncertain. For expanding the population benefit from PD-1/PD-L1 blocking therapy, the issue is worthy of further interrogation and investigation. Besides, the combinational treatment could slow down the tumor growth and provide more time for patients to receive treatment, further surgery or even radiation therapy is still warranted.

In conclusion, lactate/GPR81 pathway blockade and metformin synergistically inhibited colorectal cancer cell growth and activated CD8^+^ T cells. In addition, the combination simultaneously decreased glycolysis and OXPHOS, which might result from the down-regulation of glycolytic enzymes, mTOR phosphorylation, and GPR81 levels, but not glucose uptake. And it also significantly reduced serum lactate levels. In addition, the therapeutic effect was further improved in combination with a PD-1/PD-L1 blockade via significantly increasing the number of CD8^+^ T cells in the tumors and elevating the secretion of IFN-γ in the lymph nodes. In summary, our study provided a potential strategy for improving the cancer-therapeutic effect of metformin. Moreover, it may offer an effective method to raise patients’ response to the treatment of PD-1/PD-L1 blockade.

## Data Availability

The data generated during and/or analyzed during the current study are available from the corresponding author on reasonable request.

## Supplementary Materials

The following are available online at https://www.mdpi.com/article/10.3390/biom11091373/s1, Figure S1: Effect of metformin or 3-OBA on colorectal cancer cells, Figure S2: Mechanism of the combination of 3-OBA and metformin mediated metabolism inhibition, Figure S3: The combinational effects on PD-1 expression and IFN-γ secretion, Table S1: Primer sequences used for quantitative real-time PCR.
