# Supplementary material for: Dual Blockade of Lactate/GPR81 and PD-1/PD-L1 Pathways Enhances the Anti-Tumor Effects of Metformin
Source: Biomolecules. 2021 Sep 17;11(9):1373. doi: 10.3390/biom11091373 (PMC8466555; doi:10.3390/biom11091373)
Supplement: Supplementary file 1 [file biomolecules-11-01373-s001.zip › supplementary materials.pdf]

Supplementary materials

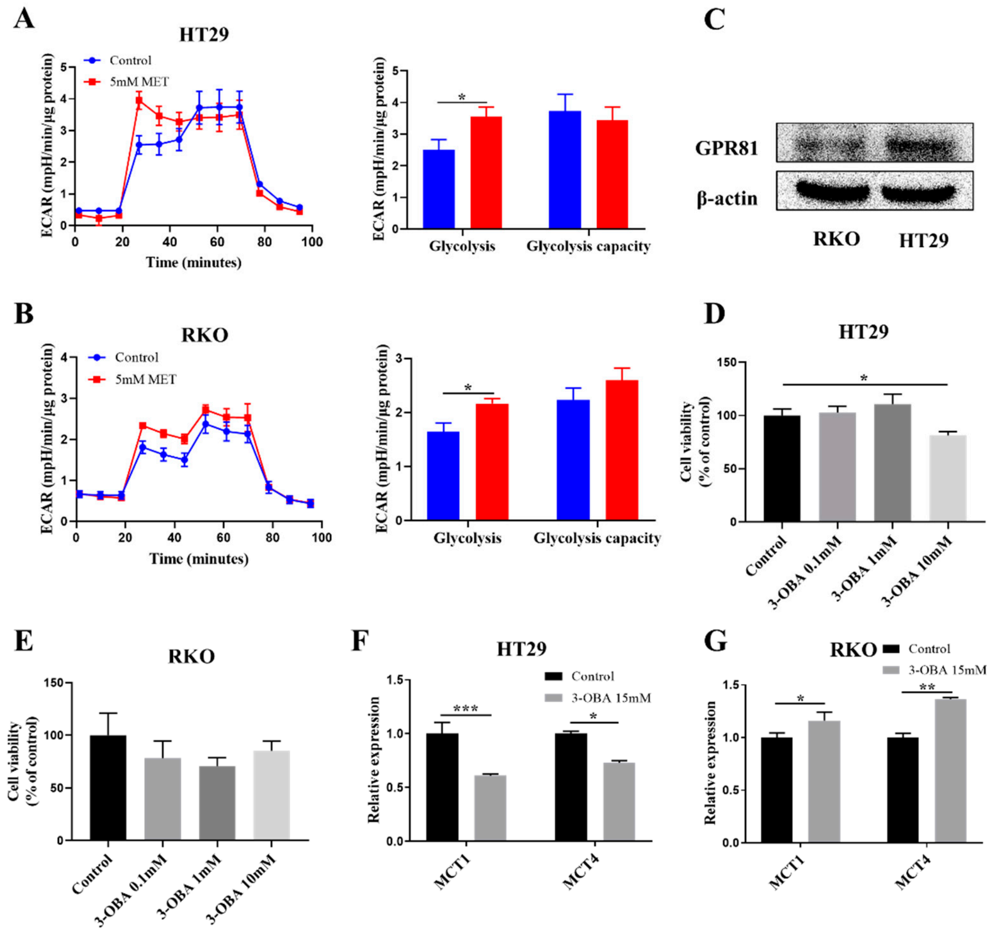

Figure S1. Effect of metformin or 3-OBA on colorectal cancer cells. (A, B) ECAR of HT29 (A) and RKO (B) cells pretreated with 5 mM metformin was detected in the presence of glucose, oligomycin (OM) and 2-deoxy glucose (2-DG). (C) The protein levels of GPR81 on RKO and HT29 cells. (D, E) Cell viability of HT29 (D) and RKO (E) cells after treatment of 3-OBA (0.1 mM, 1 mM, 10 mM). (F, G) Effect of 15 mM 3-OBA on MCT1 and MCT4 expression of HT29 (F) and RKO (G) cells. Representative histogram of at least three independent experiments were shown. *$P < 0.05$, **$P < 0.01$, ***$P < 0.001$.

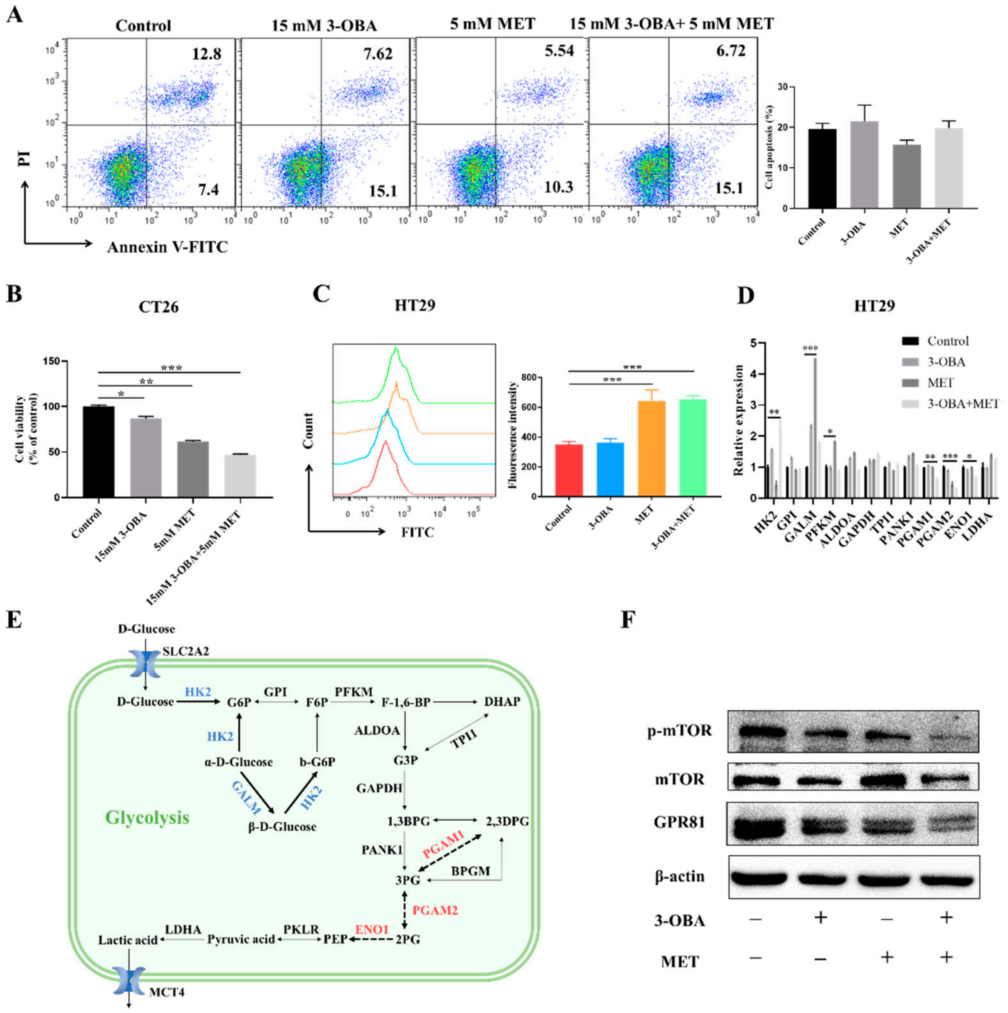

Figure S2. Mechanism of the combination of 3-OBA and metformin mediated metabolism inhibition. (A) The apoptosis assay of HT29 treated with 15 mM 3-OBA, 5 mM metformin and the combination. (B) Cell viability of CT26 treated with 15 mM 3-OBA, 5 mM metformin and the combination. (C) Glucose uptake in HT29. (D) Relative mRNA expression of glycolysis related enzymes. Representative histogram of at least three independent experiments were shown. (E) Diagram of glycolytic pathway. Abbreviations: SLC2A2, solute carrier family 2 member 2; G6P, glucose-6-phosphate; F6P, fructose-6-phosphate; F-1,6-BP, fructose-1,6-bisphosphate; DHAP, dihydroxyacetone phosphate; G3P, glyceraldehyde 3-phosphate; 1,3BPG, glyceric acid 1,3-biphosphate; 3PG, 3-phosphoglyceric acid; 2PG, 2-phosphol-D-glyceric acid; PEP, phosphoenolpyruvic acid; HK2, hexokinase 2; GPI, glucose-6-phosphate isomerase; PFKM, 6-phosphofructokinase, muscle type; ALDOA, fructose-bisphosphate aldolase A; GAPDH, glyceraldehyde-3-phosphate dehydrogenase; PANK1, pantothenate kinase 1; PGAM2, phosphoglycerate mutase 2; PGAM1, phosphoglycerate mutase 1; ENO1, alpha-enolase; PKLR, pyruvate kinase isozymes R/L; LDHA, lactate dehydrogenase A; GALM, aldose 1-epimerase. (F) The protein levels of β-action, GPR81, mTOR and p-mTOR in HT29 cell treated with 15 mM 3-OBA, 5 mM metformin and the combination. *$P < 0.05$, **$P < 0.01$, ***$P < 0.001$.

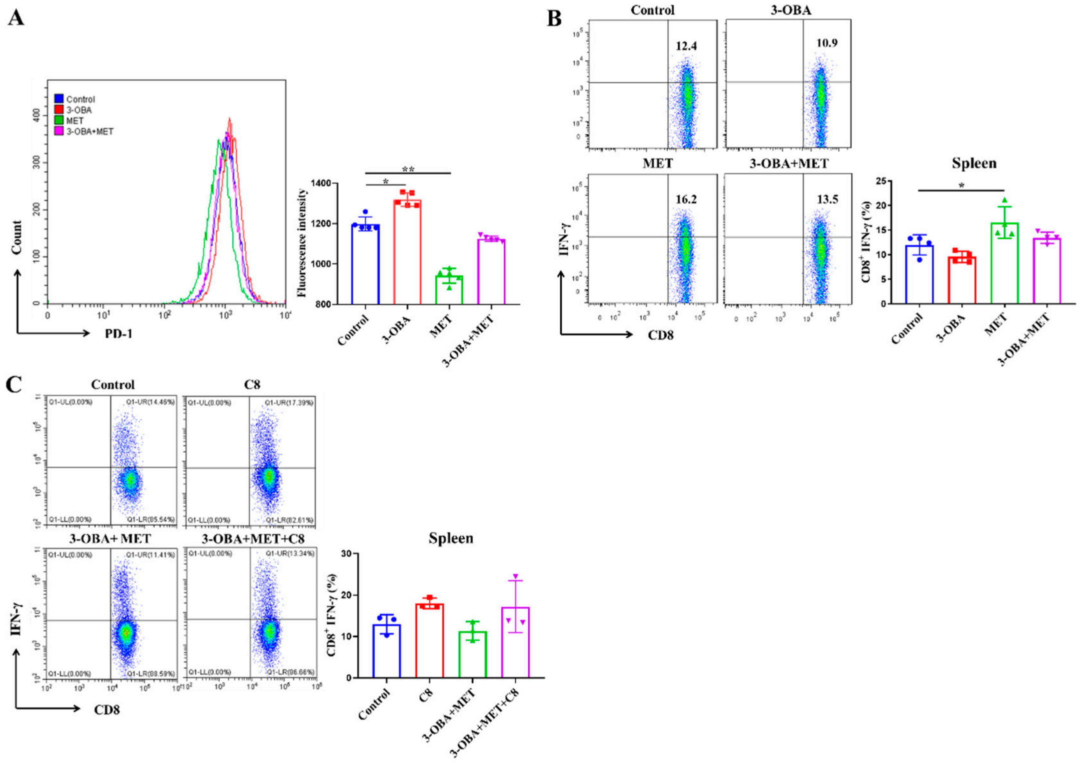

Figure S3. The combinational effects on PD-1 expression and IFN-γ secretion. (A) PD-1 expression on Jurkat cells treated with the combination of 3-OBA and metformin. The proportion of IFN-γ $CD8^+$ T cells in spleen (B) after the combined treatment of 3-OBA and metformin (C) and after the combined treatment of 3-OBA, metformin and C8. **P* < 0.05, ***P* < 0.01, ****P* < 0.001.

Table S1. Primer sequences used for quantitative real-time PCR.

Gene	*GeneBank accession number*	*Primer (5′-3′)*
GPR81	NM_032554.4	Forward: AATTTGGCCGTGGCTGATTTC Reverse: ACCGTAAGGAACACGATGCTC
HK2	NM_000189	Forward: GAGCCACCACTCACCCTACT Reverse: CCAGGCATTCGGCAATGTG
GPI	NM_001184722	Forward: CAAGGACCGCTTCAACCACTT Reverse: CCAGGATGGGTGTGTTTGACC
GALM	NM_138801	Forward: GATCACAGCCCTAGAGGTCAA Reverse: GAGGTATCCTTCCAACTCGGC
PFKM	NM_001166686	Forward: GGTGCCCGTGTCTTCTTTGT Reverse: AAGCATCATCGAAACGCTCTC
ALDOA	NM_000034	Forward: ATGCCCTACCAATATCCAGCA Reverse: GCTCCCAGTGGACTCATCTG
GAPDH	NM_001256799	Forward: GGAGTCCCTGCCACACTCA Reverse: GCCCCTCCCCTCTTCAAG
TPI1	NM_001159287	Forward: CTCATCGGCACTCTGAACG Reverse: GCGAAGTCGATATAGGCAGTAGG
PANK1	NM_138316	Forward: TGGAACGCTGGTTAAATTGGT Reverse: CCCAGTTTTCCCATAAGCAGTAT
PGAM1	NM_002629	Forward: GTGCAGAAGAGAGCGATCCG Reverse: CGGTTAGACCCCCATAGTGC
PGAM2	NM_000290	Forward: AGAAGCACCCCTACTACAACTC

		Reverse: TCTGGGGAACAATCTCCTCGT
ENO1	NM_001201483	Forward: AAAGCTGGTGCCGTTGAGAA Reverse: GGTTGTGGTAAACCTCTGCTC
LDHA	NM_001165415	Forward: ATGGCAACTCTAAAGGATCAGC Reverse: CCAACCCCAACAACTGTAATCT
